# Dissecting complex polyketide biosynthesis

**DOI:** 10.5936/csbj.201210010

**Published:** 2012-11-17

**Authors:** Patrick Caffrey

**Affiliations:** a School of Biomolecular and Biomedical Science, Centre for Synthesis and Chemical Biology, University College Dublin, Belfield, Dublin 4, Ireland

## Abstract

Numerous bioactive natural products are synthesised by modular polyketide synthases. These compounds can be made in high yield by native multienzyme assembly lines. However, formation of analogues by genetically engineered systems is often considerably less efficient. Biochemical studies on intact polyketide synthase proteins have amassed a body of knowledge that is substantial but still incomplete. Recently, the constituent enzymes have been structurally characterised as discrete domains or didomains. These recombinant proteins have been used to reconstitute single extension cycles *in vitro*. This has given further insights into how the final stereochemistry of chiral centres in polyketides is determined. In addition, this approach has revealed how domains co-operate to ensure efficient transfer of growing intermediates along the assembly line. This work is leading towards more effective re-programming of these enzymes for use in synthesis of new medicinal compounds.

## Introduction

Many drugs used in clinical medicine are based on polyketide natural products. Examples include antibiotics, anticancer agents, immunosuppressants and cholesterol-lowering statins [1]. The need for new therapeutics has motivated intensive research into the biosynthesis of these compounds. Genetic engineering of producer micro-organisms has the potential to deliver libraries of new structures. However, a better understanding of polyketide synthases (PKSs) would enable synthesis of larger and more diverse arrays, with eventual production of lead compounds in high yields at low cost.

Polyketides are assembled from activated organic acids. The process resembles fatty acid biosynthesis, in which the basic extension cycle adds a malonyl CoA derived two-carbon unit to a growing acyl chain [2]. The product is a β-ketoacyl thioester that is reduced to a β-hydroxyacyl intermediate, dehydrated to give a *trans* alkene, and reduced to give a saturated chain. Repetition of this sequence gives a long-chain fatty acid. Several enzymes catalyse this sequence of reactions. Initially the starter acetyl unit is thioester-linked to the active site cysteine thiol of the ketosynthase (KS) enzyme. An acyltransferase (AT) transfers the dicarboxylic acid extender from CoA onto the phosphopantetheine thiol of the acyl carrier protein (ACP). The KS catalyses a decarboxylative Claisen condensation to give an extended β-ketoacyl intermediate attached to the ACP. Ketoreductase (KR), dehydratase (DH) and enoylreductase (ER) enzymes catalyse the three processing steps. Chain termination is catalysed by a thioesterase (TE). Polyketide biosynthesis involves the same enzymes but generates a greater diversity of structures because the three-stage β-ketone processing sequence may be restricted, a variety of starters and extenders is used and chain lengths are more variable. Polyketide carbon chains are more functionalised than fatty acids and can undergo additional reactions such as intramolecular aldol condensation, macrolactonisation or polyether formation. Further late modifications include glycosylation, methylation, hydroxylation or halogenation [3, 4, 5].

**Figure 1:**
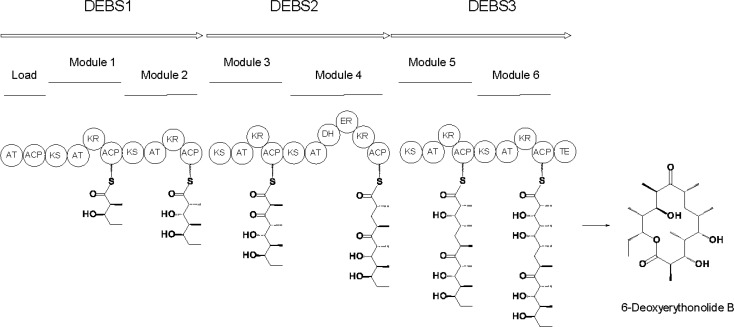
**6-Deoxyerythronolide B synthase (DEBS)**. DEBS contains a module for each of the 6 cycles of chain extension. Each module catalyses incorporation of a (2*S*)-methylmalonyl CoA derived propionate unit into an acyl chain, to form a 2-methylbranched 3-ketoacyl intermediate. The level of processing of the β-ketone group is determined by the reduction domains. Each module also determines the final stereochemistry of methyl- and hydroxyl-bearing centres.

PKSs have been classified into three main groups [1]. With type I PKSs the constituent enzymes are covalently linked within multifunctional polypeptides. Type II PKSs are composed of small discrete enzymes. Type III PKSs are KSs that build carbon chains on CoA. Type I PKSs may be iterative, with one set of biosynthetic enzymes catalysing several repetitions of an extension cycle, or modular, with multiple sets of enzymes catalysing a series of different cycles (Figure 1). The AT-less type I modular PKS subgroup use discrete ATs to acylate all of the ACP domains within the assembly line. Mixed systems composed of non-ribosomal peptide synthetase (NRPS) and PKS modules have the potential to synthesise an even greater diversity of structures [6].

Complex polyketides frequently contain alkyl groups that result from incorporation of α-branched extender units, or from the action of methyltransferases that modify the C-2 position of a growing chain. In some cases, structural complexity is increased further by β-branching, where a β-ketone group undergoes a non-decarboxylative condensation with acetyl CoA. The acetyl unit is eventually cleaved to leave a methyl group at a β position in the polyketide intermediate [7, 8]. Alkyl branching introduces chiral centres into polyketide chains, as does reduction of ketone groups to alcohols. The ability to control these enzyme systems would enable efficient production of vast arrays of diverse polyketide structures for drug discovery. The expanding knowledge of this area has been reviewed at regular intervals [9–11]. Type I, type II and type III PKSs have recently been reviewed by Hertweck [12]. This minireview will highlight some of the recent advances from biochemical studies on type I modular PKSs.

## 6-Deoxyerythronolide B synthase

The paradigm among complex PKSs is 6-deoxyerythronolide B synthase (DEBS), which makes the polyketide precursor of erythromycin A [13, 14]. The essential feature is the presence of a dedicated module for every cycle. This design allows assembly of a defined sequence of starter and extender units, variable processing of β-ketone groups, and control of methyl and alcohol stereochemistry and double bond geometry.

Early attempts at re-programming PKSs involved replacement of chromosomal coding sequences by homologous recombination within natural producer organisms. This approach is slow and laborious but is still used to investigate larger systems such as polyene PKSs [15, 16]. The first biochemical experiments relied on DEBS proteins purified from *Saccharopolyspora erythraea* [17]. Limited proteolysis was used to excise domains and didomains that could then be characterised and overexpressed as recombinant proteins [18]. PKS proteins are parallel homodimers [19]. C-terminal and N-terminal docking domains ensure that these proteins assemble into the correct order to give the final product [20]. The interactions between purified dimers are weak and overall enzymatic synthesis of 6-deoxyerythronolide B (6-dEB) *in vitro* is barely detectable [17]. A simplified system was constructed by fusing the chain-terminating TE to the C-terminus of DEBS1 [21]. This bimodular PKS catalyses efficient synthesis of triketide lactones *in vivo* and *in vitro*. The DEBS proteins and the bimodular DEBS1-TE have been expressed in heterologous hosts, *Streptomyces coelicolor* and *Escherichia coli* strains engineered for phosphopantetheinylation and precursor production. A library of over fifty 6-dEB analogues has been synthesised but in many cases yields were low [22, 23]. This represents only a fraction of the number of structures that is theoretically possible. A deeper knowledge of modular PKSs is required to exploit these enzymes more effectively.

Recombinant domains and didomains from DEBS have been characterised by x-ray crystallography and NMR spectroscopy [24–26]. These include the KS3-AT3 and KS5-AT5 didomains, and the TE, KR1, DH4 domains. NMR has been used to determine solution structures of ACP2 and ACP6 from DEBS and a fusion protein consisting of the C-terminal docking domain of DEBS1 connected to the N-terminal docking domain of DEBS 2 [27]. The structure of an ER domain from the spinosyn PKS has been determined recently [28]. The structural studies have been comprehensively reviewed in the recent past [24, 25]. KR domains are of particular interest because they influence both methyl and alcohol stereochemistry.

## Ketoreductases

PKS KR domains belong to the short-chain dehydrogenase/reductase family. The active site contains a catalytic tetrad. A Lys residue lowers the pKa of a Tyr hydroxyl group that protonates the β-ketone during hydride ion transfer. These two residues are orientated by a conserved Asn, and a Ser helps bind the β-ketone [29]. All of the active KR domains in DEBS use the 4-*pro* *S* hydrogen of NADPH [30, 31].

The stereochemical outcome of a ketoreduction depends on the type of KR. A-type KRs give 3D hydroxyl groups whereas B-type KRs give 3L [32]. For example, EryKR1 is B-type whereas Ery KR2, EryKR5, and EryKR6 are A-type (Figure 1). In this review the Cahn-Ingold-Prelog nomenclature is used. In all of the structures discussed here, 3L and 3D hydroxyl groups are 3*S* and 3*R* ^1^. Ery KR1 refers to the KR domain from module 1 of DEBS, the erythromycin PKS. Likewise, Tyl, Amp, Pic and Spn refer to KRs from the tylosin, amphotericin, picromycin and spinosyn PKSs.

B-type KRs have a characteristic Leu-Asp-Asp (LDD) motif and conserved Pro and Asn residues [32, 33]. The second aspartate residue of the LDD motif is strictly conserved [28]. A-type KRs lack these motifs but contain a conserved Trp residue. Most DH domains are paired with B-type KRs and give *trans* enoyl intermediates [32]. This suggests that these double bonds are formed by dehydration of a (3*R*)-3-hydroxyacyl chains, as is the case in fatty acid biosynthesis. Some A- and B-type KRs occur in modules that incorporate methylmalonyl CoA derived extender units. These have been subdivided further according to whether they act on (2*R*)- or (2*S*)-2-methyl-3-ketoacyl substrates (see below).

The activities of discrete recombinant KR domains have been tested using the model thioester substrate (2*RS*)-2-methyl-3-ketopentanoyl N-acetylcysteamine (NAC, a surrogate for pantetheine) [34]. It is necessary to use racemic mixtures because spontaneous epimerisation of the methyl-branched centre is rapid in aqueous solution (the half-life for proton exchange in ethyl 2-methylacetoacetate, a related β-ketoester, was 4.7 min [35]). Some KRs (EryKR2, EryKR5, EryKR6) generated mixtures of up to 3 of the 4 possible stereoisomers of the 2-methyl-3-hydroxypentanoyl chain, with the “correct” product making up only a minor component of the mixture. Others (EryKR1, TylKR1 and AmpKR2) formed reduced products with the same methyl and alcohol stereochemistry as their counterparts embedded in intact PKSs [34, 36].

Saturation mutagenesis of the stereospecificity motifs was carried out by Leadlay and co-workers [37, 38]. Mutagenesis of the Leu-Asp-Asp, Pro and Asn residues in Ery KR1 gave a switch in stereospecificity with (2*RS*)-2-methyl-3-ketopentanoyl -NAC or -pantetheine thioester substrates; the product was (2*S*, 3*S*) rather than (2*S*, 3*R*) but activity decreased approximately 5-fold. However, when the mutated KR was transplanted into intact DEBS1-TE the parent stereospecificity was observed, with the lactone of a (2*S*, 3*R*)-triketide being formed [39]. While these motifs are valuable for predicting the stereospecificity of a KR they do not *per se* determine stereochemical outcome. The structure of the active site appears to be more important.

The actions of some KRs on unnatural NAC or pantetheine thioesters are unpredictable. They may show little selectivity towards the substrate with the correct methyl stereochemistry and may also fail to carry out stereospecific ketoreduction. On the other hand, other KRs have potential as catalysts for stereospecific reduction of ketones in organic synthesis [40, 41].

A high-resolution crystal structure was first presented for Ery KR1 [42]. The structure revealed that the N-terminal region, previously regarded as an AT-KR interdomain linker, stabilises the C-terminal domain that has catalytic activity. An ER domain, when present in the reduction loop, is located between the structural and catalytic domains. Structures are now available for several other KRs [43, 36, 44, 45]. The active site residues and the NADPH substrate have the same orientation in A- and B-type KRs, with the 4-*pro*-*S*-hydrogen of the nicotinamide ring positioned toward the catalytic tyrosine. The Leu-Asp-Asp and Trp motifs are on opposite sides of the catalytic groove. The polyketide substrate is thought to enter the active site from opposite directions in A- and B-type KRs. The two stereochemical outcomes result from presentation of different faces of the β-ketone group to the bound NADPH.

## Stereochemistry of condensation and ketoreduction steps

**Figure 2:**
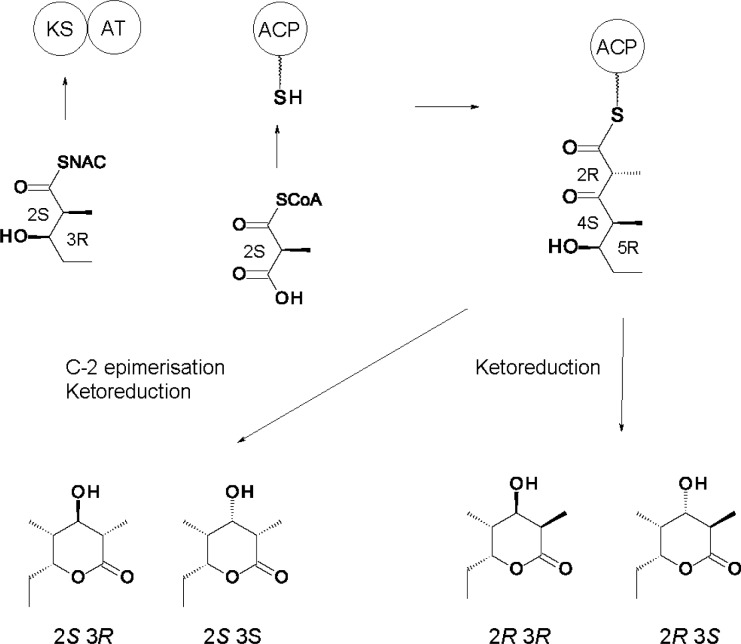
**Synthesis of triketide lactones using discrete ACPs and KS-AT didomains**. (2*S*, 3*R*)-2-methyl-3-hydroxypentanoyl-NAC is used to acylate the KS domain. The AT uses (2*S*)-methylmalonyl CoA to load the ACP. Condensation proceeds with inversion to give a (2*R*)-2-methyl-3-ketoacyl-ACP. Triketide lactone products are identified by GC-MS. Reduction of the ketone with borohydride prior to chain release gives a racemic mixture of (3*R*)- and (3*S*)- alcohols but fixes the (2*R*) methyl stereochemistry. Stereospecific ketoreduction can be achieved by adding a KR and NADPH.

Early studies revealed that the AT domains of all six DEBS extension modules use the (2*S*) stereoisomer of methylmalonyl CoA to acylate their ACPs [46]. Condensation occurs with inversion of stereochemistry at the methyl-branched carbon atom, so the initial product is (2*R*)-2-methyl-3-ketoacyl-ACP [47]. Some modules epimerise this intermediate to the (2*S*)-stereoisomer before the next step. Once structural biology revealed exact domain boundaries, Khosla and Cane were able to reconstitute polyketide synthesis using active discrete KS, AT, ACP, KR and DH enzymes [48]. These domains co-operate *in vitro* to catalyse one cycle of chain extension. KS-AT didomains are considerably more active than separated KS and AT domains. In these systems (Figure 2) the AT loads the holo-ACP domain with a (2*S*)-methylmalonyl extender from CoA. Alternatively an apo-ACP can be modified with malonyl-pantetheine by a phosphopantetheinyltransferase. The KS component of the KS-AT didomain is primed with a synthetic (2*S*, 3*R*) 2-methyl-3-hydroxypentanoyl chain that spontaneously migrates from NAC onto the active site cysteine. This diketide is then extended. The β-ketone group is reduced by inclusion of a KR domain and NADPH. Reduction fixes the C-2 methyl stereochemistry and prevents subsequent epimerisation. The ACP-bound triketide products are released by base-catalysed hydrolysis and allowed to cyclise. Trimethylsilyl ethers of the resulting lactones are analysed by a sensitive GC-MS assay. This method separates the 4 stereoisomers of the 2, 4-dimethyl-3, 5-dihydroxyheptanoic acid lactone that can form [(2*R*, 3*S*, 4*S*, 5*R*), (2*R*, 3*R*, 4*S*, 5*R*), (2*S*, 3*R*, 4*S*, 5*R*) or (2*S*, 3*S*, 4*S*, 5*R*)]. The products of a reaction mixture are identified and quantified by comparison with synthetic standards [35].

In 2-methyl-3-ketoacyl thioester intermediates, spontaneous epimerisation of the methyl-branched centre should be facile because of the acidity of the C-2 proton. Early proposals suggested that epimerising modules can provide rapidly interconverting (2*R*)- and (2*S*)-2-methyl-3-ketoacyl-ACPs with only the (2*S*)- stereoisomers being selected by downstream enzymes. However, later work with the *in vitro* system revealed that KS3-AT3 and ACP3 form a (2*R*)*-*2-methyl branched product that remains stable as long as it is attached to ACP3 [34]. It is thought that the hydrophobic cleft of the ACP protects the chain from water or prevents the β-ketone and thioester carbonyl groups from adopting a co-planar conformation. While the reconstituted module 3 generates a (2*R*)-2-methyl-3-ketoacyl-ACP3 thioester initially, the centre bearing this C-2 methyl group has the opposite stereochemistry in 6-dEB. This shows that C-2 epimerisation occurs after condensation.

There is now evidence that some KR domains catalyse both C-2 epimerisation and 3-ketoreduction and thereby impose both the methyl and the alcohol stereochemistry. Cane, Khosla and co-workers overexpressed KS-AT didomains and ACP domains from various C-2 epimerising and non-epimerising modules. Several discrete KRs were also used (Ery KR1, Ery KR6, Tyl KR1, Pic KR1) [49]. The reaction mixtures contained various combinations of KS-AT, ACP and KR proteins along with propionyl-NAC, methylmalonyl CoA and NADPH substrates. Reduced diketide acid products were analysed by chiral GC MS.

**Figure 3:**
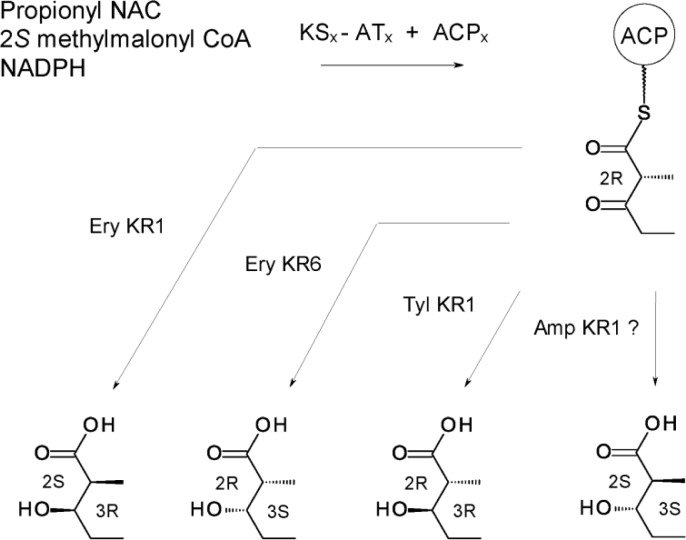
***In vitro*** **synthesis of diketides**. The combinations of KS-AT and ACP used were DEBS KS1-AT1 + ACP1 (epimerising), DEBS KS3-AT3 + ACP3 (epimerising), DEBS KS6-AT6 + ACP6 (non-epimerising), and PICS KS1-AT1 + PICS ACP1 (epimerising). All combinations gave a (2*R*)-2-methyl-3-ketoacyl-ACP initially. Enzymatic ketoreduction led to formation of a 2-methyl-3-hydroxyacyl chain with the alcohol and methyl stereochemistry characteristic of the module from which the KR was derived.

The KR domains showed increased specificity when provided with 3-ketoacyl-ACP rather than NAC thioester substrates. Ery KR1 gives a (2*S*, 3*R*) product, the same as DEBS module 1; Ery KR6 gives (2*R*, 3*S*), as does DEBS module 6; Tyl KR1 gives (2*R*, 3*R*) as does its parent module (Figure 3). Presumably Amp KR1 or Amp KR11 would give (2*S*, 3*S*) although this has not been tested. These results indicate that when a module incorporates a propionate extender unit and reduces the ketone to an alcohol, the KR decides both methyl and alcohol stereochemistry. Regardless of the source of the KS-AT and ACP, the initial product is a (2*R*)-2-methyl-3-ketoacyl-ACP. Some KRs reduce this directly whereas others apparently catalyse C-2 epimerisation (to give a (2*S*)*-*2-methyl-3-ketoacyl-ACP) prior to ketoreduction, which fixes the methyl and alcohol stereochemistry. It is less clear how an epimerised C-2 is maintained in cases where the β-ketone group is not reduced. Modules generating (2*S*)*-*2-methyl-3-ketoacyl-ACP final products invariably contain defective KR° domains. It has been proposed that these domains are inactive in ketoreduction but still catalyse C-2 epimerisation, with only the epimerised (2*S*)- form being accepted and fixed by the KS of the next module [43]. Ery KR3° is an example.

A- and B-type KRs that act on 2-methyl-branched substrates have been divided into sub-groups according to the type of module in which they appear: A1 (not C-2 epimerising) and A2 (C-2 epimerising), B1 (not C-2 epimerising) and B2 (C-2 epimerising) [43]. C-type KRs are reductase-incompetent. Those that encounter 2-methyl substrates may occur in modules that are C-2 epimerising (C2 type) or not (C1 type). Structures are now available for Ery KR1 (B2), Tyl KR1 (B1), Amp KR2 (A1), Amp KR11 (A2), Pic KR3 (C2) and Ery KR3 (C2) [36, 42–45]. Models for the structural basis of C-2 epimerisation have been discussed [43, 50]. The proposed mechanism involves removal of the α-proton, formation of an α-β enolate and re-protonation from the opposite side to invert the configuration of the methyl group. Fingerprint motifs have been proposed for predicting methyl stereochemistry as well as alcohol stereochemistry [43]. In the case of the Amp KR2 (A1 type), mutagenesis of one of the fingerprint residues Gln364 to His gave an enzyme that converted (2*RS*)-2-methyl-3-ketopentanoyl-NAC substrates to 54% (2*S*, 3*S*) and 46% (2*R*, 3*S*) products rather than 100% (2*R*, 3*S*) products [44]. An additional substitution Gly335Thr resulted in production of 94% (2*S*, 3*S*) diketides, essentially converting an A1 KR to an A2 [45]. Whether these mutations are sufficient to reverse methyl stereochemistry imposed by a complete PKS module remains to be seen. Mutagenesis of the fingerprint His to Gln in the A2-type AmphKR1 reduced activity to 6% but did not change the stereochemistry of NAC products.

It has not been possible to demonstrate that recombinant Ery KR3° epimerises (2*R*)-2-methyl-3-ketopentanoyl-ACP thioesters formed by KS3-AT3 and ACP3 in the *in* *vitro* system [35]. However, there is evidence that the KS downstream of a C2 KR is specific for a (2*S*)-2-methyl-3-ketoacyl chain. Deletion of KR3° from DEBS resulted in termination after extension cycle 3 and formation of a tetraketide [44]. This is consistent with the hypothesis that in the absence of KR3°, an un-epimerised (2*R*)-2-methyl-3-ketoacyl chain is formed that is not extended by KS4. The active site Tyr and Ser residues may allow alignment of the β-ketone and thioester carbonyls and thereby facilitate epimerisation. Mutagenesis of both of these residues to Phe led to production of both the tetraketide and 6-dEB. This suggested that the rate of epimerisation had been reduced [44].

PKS reactions that determine stereochemistry involve acyl-ACP substrates. There is increasing evidence that ACP domains make an important contribution to stereochemical outcomes. (2*RS*)*-*2-methyl-3-ketopentanoyl-NAC thioesters were used to prime triketide formation by a system composed of module 2 of the nanchangamycin synthase (NANS) fused to a TE domain [51]. Both the (2*R*)- and the (2*S*)- isomers were accepted by KS2 and extended with a methylmalonyl unit to form two triketide lactones. However, when the two primers were delivered from NANS ACP1 thioesters, only the (2*S*)*-*stereoisomer was accepted and extended. The K_m_ for the ACP donor was 300 times lower than that for the NAC thioester. This is further evidence that ACP_n_-KS_n + 1_ interactions are important in determining methyl stereochemistry, particularly where the C-2 epimerisation is not followed by ketoreduction [51].

While the last module of the pikromycin PKS forms an unreduced (2*R*)-2-methyl-3-ketoacyl-ACP, none of the well-known PKSs forms an unreduced epimerised (2*S*)-2-methyl-3-ketoacyl-ACP thioester as a final product. A highly selective TE might be required to discriminate between (2*R*)- and (2*S*)- 2-methyl-3-ketoacyl-ACP substrates in this instance.

## Dehydratases

In fatty acid biosynthesis, DH enzymes act on (3*R*)-3-hydroxyacyl-ACP thioesters and catalyse a *syn* elimination of water to give a *trans* double bond [52]. In PKSs most DH domains give *trans* double bonds and are paired with B-type KRs that give (3*R*)-3-hydroxyacyl substrates. Inactivation of one of these DH domains should leave an alcohol of known stereochemistry. This has been verified experimentally for module 2 of the picromycin PKS [53].

The DH4 domain of DEBS was shown to act only on (2*R*, 3*R*)-2-methyl-3-hydoxypentanoyl-ACP4 to catalyse a *syn* elimination of water to form trans 2-methyl-pent-2-enoyl-ACP. The other three stereoisomers ((2*S*, 3*R*), (2*S*, 3*S*), (2*R*, 3*S*)) were not dehydrated [54]. The reverse reaction was also demonstrated. The reaction equilibrium favours the 3-hydroxyl form; however, the dehydrated form is rapidly consumed by the ER or the KS of the next module. The DH2 of the nanchangamycin PKS was also shown to act on a (2*R*, 3*R*)-2-methyl-3-hydoxyacyl-ACP substrate to form a trans double bond [55].

A DH catalysing a *syn* elimination of water from a (3*S*)- 3-hydroxyacyl chain would be expected to give a *cis* double bond. Some of the rare *cis* double bonds in complex polyketides appear to result from such dehydration reactions. Plm1 and Plm2 from the phoslactomycin PKS and CurJ from the curacin NRPS-PKS are all unimodular proteins that contain DH domains paired with A-type KRs (the conserved Trp is present in all 3 whereas the Leu-Asp-Asp, Pro and Asn residues are absent) [56, 57]. All three modules generate *cis* double bonds. Reynolds and co-workers obtained experimental evidence that Plm1 imposes the double bond geometry. A *cis* enoyl chain was extended by Plm2 whereas the *trans* isomer was not incorporated intact [56]. Other *cis* double bonds in polyketides appear to arise from a different route as a late step, an example is found in borrelidin [58, 52]. A third *cis* double bond in phospholactomycin results from elimination of water as a post PKS modification [59].

## Enoyl reduction

In fatty acid biosynthesis NADPH is used to reduce a trans enoyl-ACP to a saturated acyl-chain. In principle, the hydride ion can be added to either face of C-3 and either side of C-2 can be protonated. There are four possible stereochemical routes, all of which are used by different FAS ERs from various organisms [51]. In cycle 4 of erythromycin biosynthesis, the (2*R*)-2-methyl-3-ketoacyl intermediate is reduced to a (2*R*, 3*R*)-2-methyl-3-hydroxyacyl chain that is dehydrated to a *trans* 2-methyl-α-β-unsaturated acyl-thioester. This is then reduced by Ery ER4 to a (2*S*)*-*2-methyl-branched acyl chain. The RAPS ER13 acts on a similar substrate but gives a (2*R*)*-*2-methyl-branched chain.

Leadlay and co-workers have investigated the stereochemistry of enoyl reduction [60]. A triketide lactone synthase with a complete reduction loop (DH-ER-KR) in module 2 was used to test different ER domains. A conserved Tyr (residue 51 with numbering from the domain start) was identified in ER domains giving (2*S*)-2-methyl-branched products. Mutagenesis of this residue Tyr51→Val resulted in a switch of methyl stereochemistry by the DEBS ER4 domain. However, the corresponding Val→Tyr mutation in the RAPS ER13 did not give the reverse switch from (2*R*) to (2*S*) products. Six other conserved residues were replaced by site-directed mutagenesis [61]. None was found to be indispensable, but Lys236 may have a role in stereospecificity. One sixth of the triketide lactone made by the Lys236Ala mutant had reversed methyl stereochemistry at C-2. This suggests that protonation may occur from the wrong side in the absence of the Lys236 side chain.

The ERs of PKSs and FASs belong to the medium chain dehydrogenase/reductase (MDR) superfamily. Keatinge-Clay and co-workers have recently crystallised a KR-ER didomain from the spinosyn PKS [28]. The Spn KR and ER are structurally similar to their counterparts in mammalian FAS but share a different interface with a greater surface area. The isolated ER domain is monomeric. In PKSs the KS, DH, TE and N- and C-terminal docking domains are dimeric, the AT, KR and ACP domains are monomeric. This is in contrast to mammalian FASs, in which the ER domain is dimeric and the TE domain is monomeric. The PKS reduction loop is different because the ACP has to access all of the enzymes within the module despite being restricted by a dimeric element at the C-terminus. This constraint does not apply to mammalian FASs. Unlike KR domains, the ER uses the 4 pro *R* hydride ion of NADPH. The structure revealed that the Tyr51 and Lys236 residues examined by the Leadlay group are within 5 A° of the cofactor, along with a conserved Asp residue.

## ACP-KS interactions

Mixing various KS-AT didomains with various ACP domains revealed that some combinations were more active than others in catalysing chain extension *in vitro*. KS domains show some preferences towards ACP domains. KS3 works with ACP3 and ACP5, less well with ACP2 or 4 and poorly with ACP1 or 6 [62, 48]. ACP-KS interactions have been investigated by biochemical and computational methods.

ACP domains are composed of three helices separated by two loops. Phosphopantetheinyltransferases modify the Ser within the motif GXDSL, which is located at the N-terminus of helix II. Studies with ACP3/ACP6 hybrids revealed that loop I determines whether the ACP recognises KS3-AT3 or KS6-AT6 in the *in vitro* chain elongation assay [63]. Modelling of this interaction indicates that the ACP interacts with a deep cleft formed by the KS, the KS-AT interdomain linker and AT. Mutagenesis of loop 1 revealed that residues 44 and 45 are important for molecular recognition by the KS-AT didomain. Further modelling indicates that these residues interact electrostatically with two sites within the KS-AT linker. Within a homodimeric PKS module, the ACP domain docks with the same polypeptide chain but co-operates with the KS in the opposing subunit to catalyse an elongation. A hydrophobic patch on the KS interacts with the region surrounding the phosphopantetheinylated serine of ACP.

**Figure 4:**
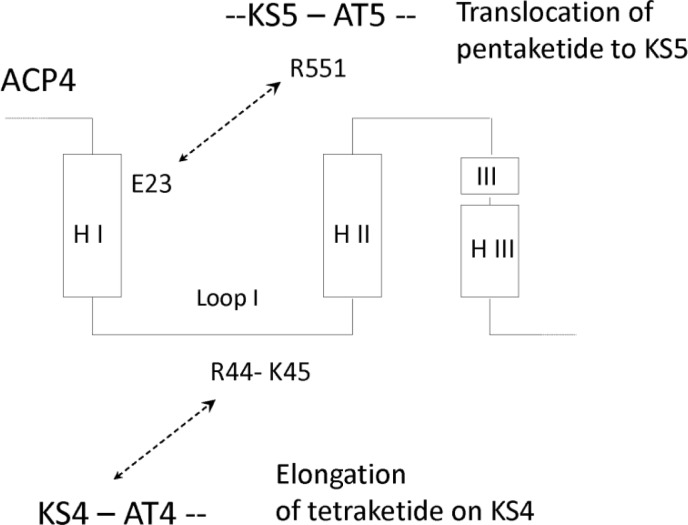
**ACP4 docking interactions for elongation and translocation**. A region of loop 1 functions in an elongation docking interaction with KS4-AT4. The N-terminal region of helix I engages in a translocation docking interaction with KS5-AT5.

**Figure 5:**
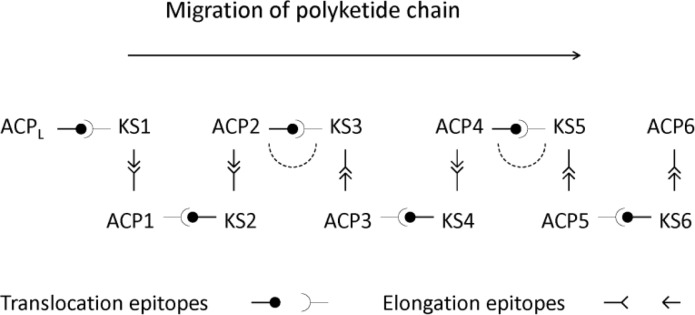
Schematic representation of KS-ACP interactions in DEBS. ACP_n_ cannot transfer an extended polyketide back to KS_n_ because their translocation epitopes are incompatible. This prevents iterative operation of a module. The elongation interactions are also electrostatic and are drawn to depict the preference of ACP3 for KS3 and KS5, the weak preference for KS2 and KS4 and the lack of co-operation with KS1 and KS6. The dashed arcs represent interdimer docking interactions between DEBS1 and DEBS2 and between DEBS2 and DEBS3.

Further mutagenesis was carried out to identify regions of the ACP that function in translocation of an extended polyketide to the next KS in the assembly line [64]. Ten residues at the N-terminus of helix I are important for transfer of a polyketide from ACP_n_ to KS_n + 1_. The residue at position 23 is critical in ACP-KS recognition for translocation. For example, during transfer from DEBS module 4 to module 5, the translocation interactions principally involve Glu23 on helix 1 of ACP4 and Arg551 in the KS5-AT5 interdomain linker (Figure 4). To effect translocation of the pentaketide, ACP4 collaborates with KS5 by docking in the same deep cleft used by ACP5 during elongation. However, the translocating ACP4 docks in a different position and orientation from that predicted for the elongating ACP5. After completion of cycle 5, the hexaketide cannot translocate back to KS5 because the ACP5 residue corresponding to position 23 is His. This does not form a translocation docking interaction with Arg551 in the KS5-AT5 interdomain region. As a general pattern, the residue at position 23 in an ACP has the same charge as residue 551 of the KS-AT interdomain within the same module and the opposite charge to the corresponding position in the downstream module. This prevents iterative operation of a module but allows forward translocation to the next KS. The model for elongation and translocation interactions between ACP and KS domains is represented schematically in Figure 5.

To test this hypothesis, Khosla and co-workers replaced helix I of ACP3 with helix I from ACP2. The hybrid ACP was tested with KS3-AT3, (2*S*, 3*R*)-2-methyl-3-hydroxypentanoyl-NAC, (2*S*)-methylmalonyl CoA, KR2 and NADPH [64]. The reconstituted module 3 catalysed two cycles of chain extension, indicating that the hybrid ACP could engage KS3 for elongation and translocation. Chain growth stopped after two cycles. The active site of KS3 may not be able to accommodate a chain length greater than a tetraketide. The KR domain only reduced the ketone formed in the first of these two cycles.

It will be interesting to see whether similar ACP-KS interactions impose unidirectional flow of extended intermediates in other PKSs.

## Conclusions

The modular nature of the erythromycin PKS was first recognised in 1990. Since that time there has been considerable progress in understanding the enzymology of complex polyketide biosynthesis. Structural and biochemical studies have revealed many of the stereochemical aspects of the process. Ketoreductase domains appear to determine methyl and alcohol stereochemistry in polyketide chains, although the precise mechanism by which some (2*R*)-2-methyl-3-ketoacyl-ACP intermediates undergo C-2 epimerisation is still unclear. Studies with recombinant discrete domains are revealing how best to achieve efficient synthesis of polyketides by artificially constructed assembly lines. This should enable the full potential of PKSs to be exploited for synthesis of libraries of new compounds.

## Footnotes

1 3D alcohols in polyketide intermediates are nearly always designated 3*R* in the CIP nomenclature system. However, since polyketides in general can have variable substituents at C-2 and C-4, a 3D alcohol may be designated 3*S* in rare cases where C-4 has a higher priority than C-2. Similarly 3L alcohols are usually 3*S* but may sometimes be designated 3*R*. In 2-methyl-3-ketoacyl-intermediates the thioester group invariably gives C-1 the highest priority so 2D methyl groups are always designated 2*R* and 2L methyl groups are always designated 2S. For all of the structures discussed in this article 3*R* hydroxyl groups are 3D and 3*S* hydroxyl groups are 3L.
